# Triglyceride glucose-body mass index and the risk of progression to diabetes from prediabetes: A 5-year cohort study in Chinese adults

**DOI:** 10.3389/fpubh.2023.1028461

**Published:** 2023-02-03

**Authors:** Yong Han, Haofei Hu, Qiming Li, Zhe Deng, Dehong Liu

**Affiliations:** ^1^Department of Emergency, Shenzhen Second People's Hospital, Shenzhen, Guangdong, China; ^2^Department of Nephrology, Shenzhen Second People's Hospital, Shenzhen, Guangdong, China

**Keywords:** triglyceride-glucose index, prediabetes, diabetes, smooth curve fitting, non-linear relationship

## Abstract

**Objective:**

Evidence regarding the relationship between the triglyceride glucose-body mass index (TyG-BMI) and the risk of progression from prediabetes to diabetes remains limited. Our study aimed to investigate the relationship between them in patients with prediabetes.

**Methods:**

In this retrospective cohort study, data were collected from 25,279 patients with prediabetes who received health checks between 2010 and 2016. We used a Cox proportional-hazards regression model to examine the relationship between TyG-BMI and diabetes risk. We used Cox proportional hazards regression with cubic spline functions and smooth curve fitting to identify the nonlinear relationship between them. In addition, A series of sensitivity and subgroup analyses were also conducted.

**Results:**

The mean age of the included participants was 49.29 ± 13.82 years old, and 1,6734 (66.2%) were male. The mean TyG-BMI was 219.47. The median follow-up time was 2.89 years, and 2,687 (10.63%) individuals had a final diagnosis of diabetes. After adjusting for covariates, TyG-BMI was positively linked with incident diabetes in patients with prediabetes (HR = 1.011, 95%CI 1.010–1.012). TyG-BMI had a non-linear connection with diabetes risk, and its inflection point was 231.66. Right and left effects sizes (HR) at the inflection point were 1.017 (95%CI:1.014–1.019) and 1.007 (95%CI:1.005–1.009), respectively. The sensitivity analysis demonstrated the robustness of these results.

**Conclusion:**

This study demonstrated a positive, non-linear relationship between the TyG-BMI and diabetes risk in Chinese patients with prediabetes. When the TyG-BMI was <231.66, there was a significant positive association between TyG-BMI and the risk of progression from prediabetes to diabetes. This study serves as a reference to promote clinical consultation and optimize diabetes prevention decisions for patients with prediabetes.

## Introduction

Diabetes mellitus (DM) is a very common complex of endocrine and metabolic disorders that afflicts hundreds of millions of people worldwide (1). According to the International Diabetes Federation Diabetes Atlas, diabetes affects 425 million people worldwide in 2017. It is estimated that the number of people with diabetes will increase to 629 million by 2045 (2). It is well known that diabetes can have long-term complications affecting the kidneys, nerves, eyes, and cardiovascular system (3–5). Besides, diabetes is a leading cause of disability and mortality (6). Therefore, diabetes is a serious health concern that imposes a heavy economic burden on societies worldwide.

Prediabetes is an intermediate stage between normoglycemia and diabetes, characterized by impaired glucose metabolism. It generally reflects the presence of either or both impaired glucose tolerance and fasting glucose. According to the International Diabetes Federation (IDF), around 374 million adults in the world had prediabetes in 2017, with a global prevalence of 7.7%. In 2045, there will be 548 million adults with prediabetes, equivalent to 8.4% of the world's population (2). In the US alone, 86 million adults aged ≥18 had prediabetes (7). The prevalence of prediabetes among adults has reached 35.7% in a nationwide cross-sectional survey in China (8). Seventy percent of those with prediabetes will eventually develop diabetes, according to an American Diabetes Association (ADA) expert panel (7). Thus, prediabetes is often viewed as a warning sign. However, most patients with prediabetes often ignore this metabolic abnormality and neglect its significance. Therefore, knowing the risk factors for progression from prediabetes to diabetes is particularly important in preventing or delaying diabetes and its complications.

It has been recognized that insulin resistance (IR) plays an essential role in many metabolic disorders, such as metabolic syndrome, non-alcoholic fatty liver disease (NAFLD), diabetes, and obesity (9–12). IR is one of the major factors in the development of diabetes, so identifying individuals with IR before diabetes develops is crucial. The hyperinsulinemic euglycemia clamp remains the gold standard for IR measurement, but it is not widely applicable in clinical practice by its labor intensity, cost, and ethical issues (13). Thus, a simple, reproducible, and reliable index for detecting IR is urgently needed. Researchers have demonstrated that the triglyceride-glucose index (TyG) index consists of the product of fasting plasma glucose (FPG) levels and triglyceride (TG) and is highly sensitive and specific for recognizing IR compared to the euglycemic hyperinsulinemic clamp test (14, 15). The triglyceride glucose-body mass index (TyG-BMI) has been developed as an obesity-related parameter in recent years. It is the product of the body mass index (BMI) and the TyG index. According to a recent study, TyG-BMI can simultaneously capture several clinical variables, such as BMI, blood glucose, and lipid profile, and more closely reflect IR than index alone (16). Since IR plays an important role in diabetes pathogenesis, we hypothesized that TyG-BMI might be a useful predictor of diabetes. Unfortunately, the current research on the relationship between diabetes and TyG-BMI is limited, with only two studies addressing the topic (17, 18). In addition, previous studies investigating the association between TyG-BMI and diabetes were for the general population. The relationship between them has not been reported in patients with prediabetes, a population at high risk of developing diabetes. Therefore, in order to determine the relationship between TyG-BMI and the risk of progression from prediabetes to diabetes, we conducted a retrospective cohort study using published data.

## Methods

### Study design

A retrospective cohort study design was used in this study, and data were collected from a Chinese computerized database by Chinese researchers (Chen et al. 19). TyG-BMI was the target-independent variable. Diabetes (DM) (dichotomous: 0 = non-DM, 1 = DM) was the outcome variable.

### Data source

The raw data were obtained free of charge from DATADRYAD (www.datadryad.org), which was provided by Chen et al. (19). Dataset was derived from a published article -association of body mass index and age with incident diabetes in Chinese adults: a population-based cohort study (https://doi.org/10.5061/dryad.ft8750v). This is an open-access article given in accordance with the Creative Commons Attribution Non Commercial (CC BY-NC 4.0) license, which enables people to share, remix, modify, and create a derivative work from this work for non-commercial purposes as long as the author and source are credited (19).

### Study population

The original researchers extracted data from a computerized database created by the Rich Healthcare Group in China, which contains all medical records for participants who received health checks in 32 regions and 11 cities between 2010 and 2016 (19). The original study was initially approved by the Rich Healthcare Group Review Board, and the data was retrieved retroactively. The institutional ethics committee did not require informed consent or approval for the retrospective study (19). Therefore, ethical approval was not required for the current secondary analysis. Furthermore, the original study was carried out in compliance with the Helsinki Declaration. So did this secondary analysis.

**Figure 1:**
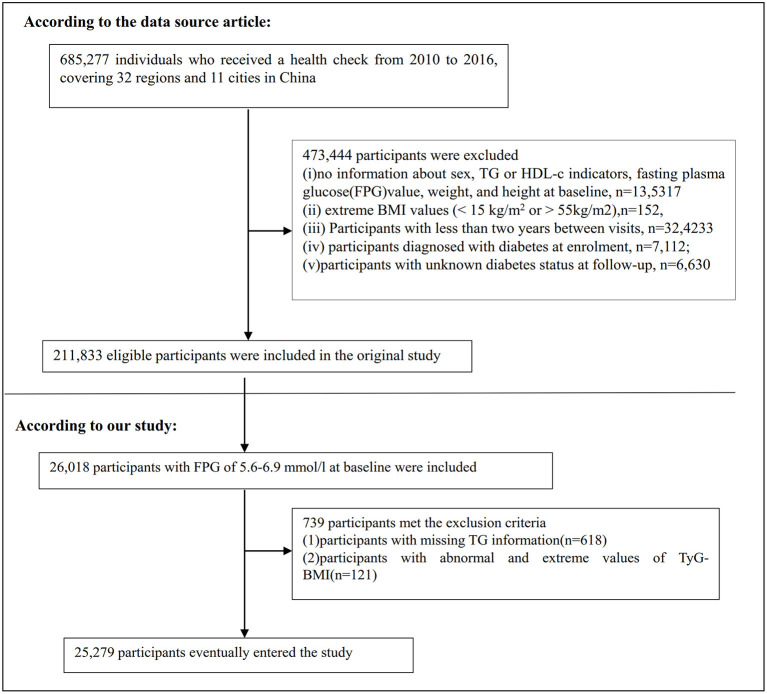
Flowchart of study participants.

685,277 participants who were at least 20 years old and had passed at least two health examinations were initially enrolled in the original study. 473,744 participants were excluded after that. Finally, the original study included 211,833 individuals in its analysis (19). Following are the exclusion criteria for the original study: (i) participants diagnosed with diabetes at enrolment; (ii) no information about FPG value, sex, height, and weight at baseline; (iii) extreme BMI values (< 15 or >55 kg/m^2^); (iv) Participants with < 2 years between visits; (v) participants with unknown diabetes status at follow-up (19). We first included 26,018 participants with baseline FPG of 5.6–6.9 mmol/l in the current study. Prediabetes is defined as an FPG level of 5.6–6.9mmol/L according to the American Diabetes Association 2021 criteria (20). After that, we excluded participants who lacked TG data (*n* = 618) and those who had abnormal or extreme TyG-BMI (greater or < 3 standard deviations from the mean) (*n* = 317). Ultimately, 25,279 participants were included in the current secondary analysis. Figure 1 shows how participants were selected.

## Variables

### Independent variable

The detailed procedure for defining TyG-BMI was as follows: TyG-BMI = BMI × TyG index, where TyG index = ln [FPG (mg/dL) × TG (mg/dL)/2] and BMI = weight / height^2^ (16). It was important to note that relevant information for TG, BMI, and FPG was obtained at baseline.

### Outcome measures

The outcome variable of interest in our investigation was incident diabetes (dichotomous variable: 1 = DM and 0 = non-DM). FPG≥7.0 mmol/L or self-report at follow-up evaluation was used to define incident diabetes (19).

### Covariates

The covariates in our study were selected according to the previous literature and our medical experience. Covariates included the following: (i) continuous variables: weight, height, age, aspartate aminotransferase (AST), blood urea nitrogen (BUN), alanine aminotransferase (ALT), serum creatinine (Scr), BMI, systolic blood pressure (SBP), diastolic blood pressure (DBP), high-density lipoprotein cholesterol (HDL-c), total cholesterol (TC), low-density lipid cholesterol (LDL-c); (ii) categorical variables: drinking status, family history of diabetes, sex, and smoking status.

### Data collection

In the initial study, qualified researchers used standardized questionnaires to gather baseline data on lifestyle (drinking and smoking status), demographics (age and sex), and family history of diabetes. Standard mercury sphygmomanometers measured blood pressure. During each visit, fasting venous blood samples were taken at least 10 h after a fast. A Beckman 5,800 autoanalyzer was used to measure plasma glucose, TC, TG, HDL-c, and LDL-c (19).

### Missing data processing

In this second analysis, the number of participants whose data are missing of SBP, DBP, ALT, Scr, BUN, LDL-c, HDL-c, AST, drinking status, and smoking status was 7 (0.03%), 7 (0.03%), 211 (0.84%), 1,119 (4.43%), 2,439 (9.65%), 9,291 (36.75%), 9,921 (39.25%), 14,118 (55.85%), 16,727 (66.17%), and 16,727 (66.17%), respectively. This study used multiple imputations for missing data to reduce the variation brought on by missing variables (21). The imputation model (type was Linear regression, iterations were 10) included sex, age, DBP, HDL-c, TC, AST, BUN, Scr, SBP, ALT, LDL-c, drinking status, family history of diabetes, and smoking status. Missing-at-random (MAR) assumptions are used in missing data analysis procedures (21, 22).

### Statistical analysis

We stratified the participants by quartiles of TyG-BMI. The means and standard deviations were presented for continuous variables with Gaussian distributions, medians were reported for skewed distributions, and percentages and frequencies were presented for categorical variables. We used the Kruskal-Wallis H-test (skewed distribution), the One-Way ANOVA test (normal distribution), or χ2 (categorical variables) to test for differences among different TyG-BMI groups.

We examined the link between TyG-BMI and diabetes risk in patients with prediabetes using univariate and multivariate Cox proportional-hazards regression models after collinearity screening. These included a non-adjusted model (no covariates adjusted), a minimally adjusted model (Model I: adjusted age and sex), and a fully adjusted model were used (Model II: adjusted age, sex, BUN, DBP, SBP, AST, ALT, HDL-c, LDL-c, drinking status, smoking status and family history of diabetes). HR and 95% confidence intervals (CI) were recorded in this study. The collinearity screening also excluded TC from the final multivariate Cox proportional hazards regression equation since it was collinear with other variables (Supplementary Table S1).

Besides, we used Cox proportional hazards regression with cubic spline functions and smooth curve fitting to explore the non-linear relationship between the TyG-BMI and diabetes risk in participants with prediabetes. Recursive algorithms were used to calculate the inflection point if non-linearity was found. Then a two-piecewise linear regression model was fitted to calculate the threshold effect of the TyG-BMI on incident diabetes according to the smoothed graph.

A stratified Cox proportional hazard regression model was used to conduct subgroup analysis across several subgroups (age, sex, SBP, smoking status, and drinking status). Firstly, the interaction test between these variables and TyG-BMI was performed before the subgroup analysis. Secondly, continuous variables, including SBP, and age, were converted into categorical variables based on clinical cut-off points (age: < 30, ≥30 to < 40, ≥40 to < 50, ≥50 to < 60, ≥60 to < 70, ≥70 years old; SBP: < 140, ≥140 mmHg) (23). Thirdly, we adjusted each stratification for all other factors (sex, age, AST, SBP, ALT, DBP, HDL-c, BUN, LDL-c, smoking status, family history of diabetes, and drinking status) besides the stratification factor itself. Ultimately, the likelihood ratio test was used to determine whether interaction terms existed in models with and without interaction terms. The likelihood ratio test compares models with and without the multiplicative interaction term(s); the log-likelihood of models with main effects was compared with the log-likelihood of models that contained main effects and the interaction terms to determine the statistical significance of interactions (24).

To test the robustness of the results, we performed a series of sensitivity analyses. We converted the TyG-BMI into a categorical variable according to the quartile and calculated the *P*-value for the trend to test the results of the TyG-BMI as a continuous variable and to explore the possibility of non-linearity. Previous studies have suggested that drinking status, family history of diabetes, and BMI are significantly related to diabetes (25–27). Therefore, we performed sensitivity analyses after excluding alcohol drinkers and smokers and participants with a family history of diabetes. Additionally, we further explored the association between the TyG-BMI and diabetes risk in participants with BMI < 25 kg/m^2^. Further, to further confirm our findings' reliability, we used a generalized additive model (GAM), which included the continuity covariate as a curve in the equation.

Finally, we construct a receiver operating characteristic (ROC) curve to estimate the ability of TyG-BMI, BMI, TyG, TG, and TG/HDL-c ratio to predict the risk of diabetes in patients with prediabetes.

All results were written according to the STROBE statement (28). Both Empower Stats (X&Y Solutions, Inc. Boston, MA, http://www.empowerstats.com) and the R statistical software packages (http://www.r-project.org, The R Foundation) were used to conduct all analyses. Statistical significance was defined as *P*-values under 0.05 (two-sided).

## Results

### Characteristics of participants

**Figure 2:**
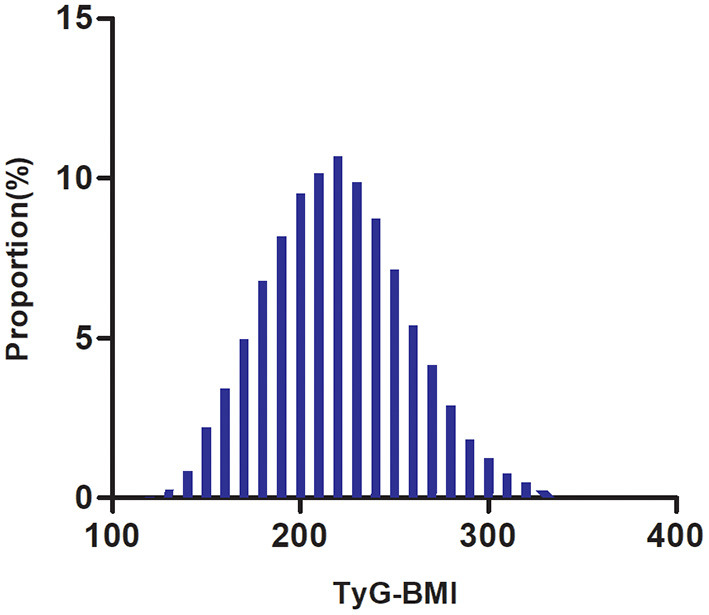
Distribution of TyG-BMI. It presented a normal distribution, ranging from 116.94 to 334.08, with a mean of 219.47.

The study participants' demographic and clinical characteristics are presented in Table 1. The mean age was 49.29 ± 13.82 years old, and 16,734 (66.2%) were male. The mean TyG-BMI was 219.47. Over a median follow-up period of 2.89 years, 2,687 (10.63%) participants developed diabetes. We divided adults into subgroups based on TyG-BMI quartiles (Q1: < 193.08, Q2: 193.08–218.21, Q3: 218.21–244.04, Q4: ≥244.04). The highest quartile (Q4: ≥244.04) showed significant increases in age, height, weight, BMI, DBP, SBP, TG, LDL-c, TC, AST, ALT, TyG-BMI, TyG, Scr, and BUN in comparison with the lowest quartile (Q1: < 193.08); however, HDL-c showed the opposite trend. Moreover, the highest quartile had a higher proportion of men, current drinkers, and current smokers. The TyG-BMI presents a normal distribution, ranging from 116. 94 to 334.08, with a mean of 219.47 (Figure 2). The presence or absence of diabetes at the last follow-up visit was used to split the participants into two groups. Supplementary Figure S1 shows the distribution of TyG-BMI for the two groups. The TyG-BMI distribution level was lower in the non-diabetes group. In contrast, the group with diabetes had a higher distribution level of the TyG-BMI.

**Table 1:** The baseline characteristics of participants.

**TyG-BMI quartile**	**Q1 (< 193.08)**	**Q2 (193.08–218.21)**	**Q3 (218.21–244.04)**	**Q4 (≥244.04)**	***P*-value**
Participants	6,319	6,320	6,320	6,320	
Age (years)	45.25 ± 14.30	50.29 ± 13.79	51.63 ± 13.34	50.00 ± 12.96	< 0.001
Height (cm)	165.18 ± 8.29	166.32 ± 8.40	167.17 ± 8.25	168.06 ± 8.16	< 0.001
Weight (kg)	57.30 ± 7.40	65.93 ± 7.50	71.97 ± 7.97	81.05 ± 9.93	< 0.001
BMI (kg/m^2^)	20.94 ± 1.66	23.77 ± 1.28	25.69 ± 1.39	28.63 ± 2.21	< 0.001
SBP (mmHg)	120.70 ± 16.57	126.39 ± 17.05	129.17 ± 17.32	132.41 ± 17.26	< 0.001
DBP (mmHg)	73.91 ± 10.09	77.40 ± 10.60	79.76 ± 10.86	82.44 ± 11.17	< 0.001
TG (mmol/L)	0.84 (0.63–1.11)	1.24 (0.96–1.65)	1.67 (1.25–2.24)	2.33 (1.70–3.34)	< 0.001
TYG	8.28 ± 0.43	8.69 ± 0.43	8.99 ± 0.46	9.38 ± 0.55	< 0.001
TyG-BMI	173.25 ± 14.59	206.08 ± 7.26	230.53 ± 7.42	267.99 ± 19.45	< 0.001
TC (mmol/L)	4.65 ± 0.88	4.93 ± 0.93	5.07 ± 0.92	5.25 ± 0.99	< 0.001
FPG (mmol/L)	5.86 ± 0.27	5.92 ± 0.30	5.97 ± 0.33	6.03 ± 0.34	< 0.001
HDL-c (mmol/L)	1.42 ± 0.31	1.34 ± 0.31	1.29 ± 0.28	1.27 ± 0.30	< 0.001
LDL-c (mmol/L)	2.68 ± 0.68	2.90 ± 0.71	2.96 ± 0.71	3.00 ± 0.75	< 0.001
ALT (U/L)	15.50 (12.00–21.30)	20.00 (15.00–28.00)	24.50 (18.00–35.60)	32.00 (22.30–48.30)	< 0.001
AST (U/L)	22.47 ± 8.95	24.65 ± 11.10	27.02 ± 10.51	31.18 ± 14.62	< 0.001
BUN (mmol/L)	4.82 ± 1.25	5.00 ± 1.23	5.07 ± 1.26	5.06 ± 1.25	< 0.001
Scr (μmol/L)	68.32 ± 15.32	72.37 ± 15.49	74.45 ± 16.42	75.80 ± 15.69	< 0.001
**Sex**					< 0.001
Male	3,092 (48.93%)	4,067 (64.35%)	4,586 (72.56%)	4,989 (78.94%)	
Female	3,227 (51.07%)	2,253 (35.65%)	1,734 (27.44%)	1,331 (21.06%)	
**Smoking status**					< 0.001
Current smoker	880 (13.93%)	1,324 (20.95%)	1,600 (25.32%)	1,911 (30.24%)	
Ever smoker	208 (3.29%)	252 (3.99%)	271 (4.29%)	303 (4.79%)	
Never smoker	5,231 (82.78%)	4,744 (75.06%)	4,449 (70.40%)	4,106 (64.97%)	
**Drinking status**					< 0.001
Current drinker	145 (2.29%)	195 (3.09%)	222 (3.51%)	362 (5.73%)	
Ever drinker	714 (11.30%)	968 (15.32%)	1,028 (16.27%)	1,161 (18.37%)	
Never drinker	5,460 (86.41%)	5,157 (81.60%)	5,070 (80.22%)	4,797 (75.90%)	
**Family history of diabetes**					0.220
No	6,177 (97.75%)	6,148 (97.28%)	6,175 (97.71%)	6,154 (97.37%)	
Yes	142 (2.25%)	172 (2.72%)	145 (2.29%)	166 (2.63%)	

Continuous variables were summarized as mean (SD) or medians (quartile interval); categorical variables were displayed as percentage (%).

FPG, fasting plasma glucose; DBP, diastolic blood pressure; BMI, body mass index; TC, total cholesterol; TG, triglyceride; SBP, systolic blood pressure; TyG, the triglyceride-glucose index; TyG-BMI, triglyceride glucose-body mass index; AST, aspartate aminotransferase; ALT, alanine aminotransferase; LDL-C, low-density lipid cholesterol; BUN, blood urea nitrogen; Scr, serum creatinine. HDL-c, high-density lipoprotein cholesterol.

### The incidence rate of diabetes in patients with prediabetes

**Figure 3:**
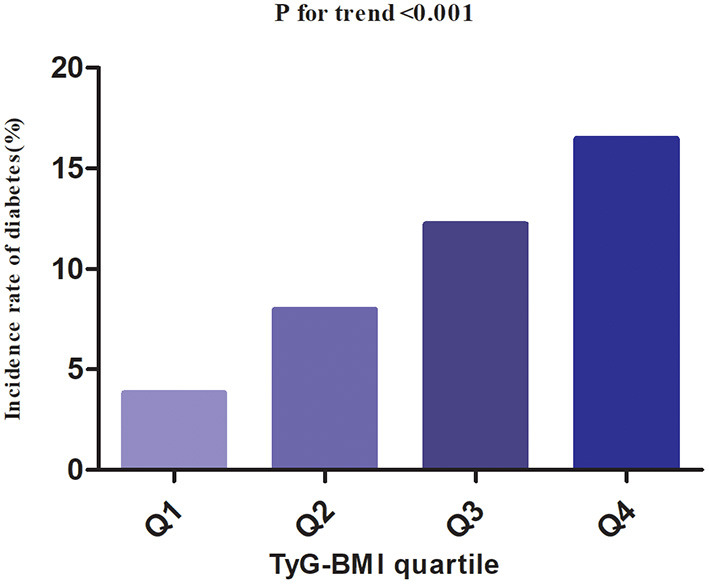
The incidence rate for diabetes according to the quartiles of TyG-BMI. Participants with the highest TyG-BMI(Q4) had higher diabetes incidence rates than those with the lowest TyG-BMI(Q1) (*p* < 0.001 for trend).

Among participants with prediabetes, 2,687 (10.63%) individuals developed diabetes. Specifically, the incidence rate of diabetes among participants with prediabetes in the TyG-BMI quartiles was 13.16, 27.09, 41.11, and 55.22 per 1,000 person-years, respectively. During a median follow-up period of 2.89 years, the overall cumulative incidence of diabetes was 11.46%, and the cumulative incidences of diabetes in each TyG-BMI quartile were Q1: 3.83%, Q2: 7.97%, Q3: 12.23%, and Q4: 16.47% (Figure 3). There was a higher incidence of diabetes among those with the highest TyG-BMI (Q4) compared to those with the lowest TyG-BMI (Q1) (*p* < 0.001 for trend) (Table 2, Figure 3).

**Table 2:** Incidence rate of diabetes in participants with prediabetes (% or Per 1,000 person-year).

**TyG-BMI**	**Participants (*n*)**	**Diabetes events (*n*)**	**Incidence rate (95% CI) (%)**	**Per 1,000 person-year**
Total	25,279	2,560	10.13(9.76–10.50)	34.27
Q1(< 193.08)	6,319	242	3.83(3.36–4.30)	13.16
Q2 (193.08–218.21)	6,320	504	7.97(7.70–8.34)	27.09
Q3 (218.21–244.04)	6,320	773	12.23(11.42–13.04)	41.11
Q4 (≥244.04)	6,320	1,041	16.47(15.56–17.39)	55.22
*P* for trend			< 0.001	

TyG-BMI, triglyceride glucose-body mass index; CI, confidence interval.

**Figure 4:**
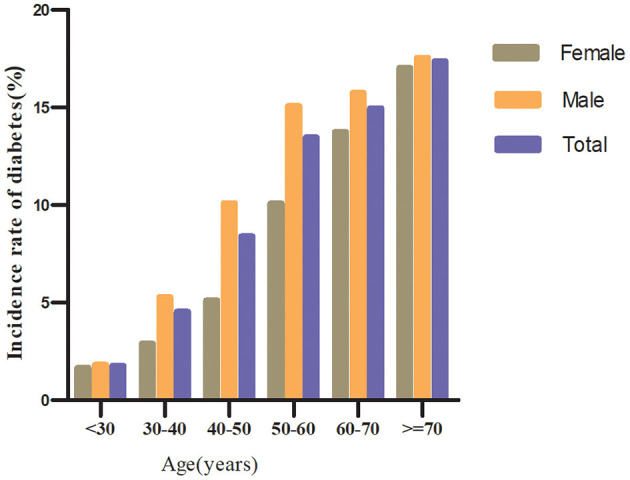
The incidence of diabetes in prediabetic patients of age stratification by 10 intervals. It shows that the incidence of diabetes in participants with prediabetes was higher in men than in women, regardless of age group. It was also found that the incidence of diabetes increased with age in both men and women.

Regardless of their age groups, men with prediabetes were more likely to develop diabetes than women in the age stratification by ten intervals (Figure 4). In addition, both males and females showed an increase in diabetes incidence with age.

### Factors influencing the risk of diabetes in patients with prediabetes analyzed by univariate Cox proportional hazards regression

Based on univariate analyses, the risk of progression to diabetes from prediabetes was not related to Scr (*P* > 0.05), but was positively correlated with age, DBP, BMI, SBP, AST, TG, ALT, TyG-BMI, TC, FPG, LDL-c, BUN, family history of diabetes, and current drinking (all *P* < 0.05; Table 3).

**Table 3:** Factors influencing the risk of diabetes in patients with prediabetes analyzed by univariate Cox proportional hazards regression.

**Exposure**	**Characteristics**	**HR (95%CI)**	***P*-value**
Age (years)	49.292 ± 13.819	1.031 (1.028, 1.034)	< 0.001
**Sex**			
Male	16,734 (66.197%)	Ref	
Female	8,545 (33.803%)	0.835 (0.766, 0.910)	< 0.001
BMI (kg/m^2^)	24.758 ± 3.265	1.119 (1.106, 1.132)	< 0.001
SBP (mmHg)	127.169 ± 17.586	1.015 (1.013, 1.017)	< 0.001
DBP (mmHg)	78.377 ± 11.135	1.017 (1.014, 1.020)	< 0.001
FPG (mmol/L)	5.945 ± 0.317	9.796 (8.889, 10.795)	< 0.001
TG (mmol/L)	1.410 (0.960–2.110)	1.116 (1.096, 1.137)	< 0.001
TyG	8.835 ± 0.619	1.800 (1.700, 1.907)	< 0.001
TyG-BMI	219.465 ± 37.051	1.012 (1.011, 1.013)	< 0.001
TC (mmol/L)	4.975 ± 0.957	1.082 (1.040, 1.125)	< 0.001
HDL-c(mmol/L)	1.330 ± 0.303	1.246 (1.097, 1.416)	< 0.001
LDL-c (mmol/L)	2.886 ± 0.724	1.064 (1.010, 1.122)	0.021
ALT (U/L)	22.000 (15.400–33.000)	1.005 (1.004, 1.006)	< 0.001
AST (U/L)	26.328 ± 11.927	1.011 (1.009, 1.012)	< 0.001
BUN (mmol/L)	4.991 ± 1.251	1.051 (1.019, 1.083)	0.002
Scr (μmol/L)	72.735 ± 15.988	1.001 (0.999, 1.004)	0.392
**Smoking status**			
Current smoker	5,715 (22.608%)	Ref	
Ever smoker	1,034 (4.090%)	1.059 (0.878, 1.276)	0.550
Never smoker	18,530 (73.302%)	0.823 (0.754, 0.899)	< 0.001
**Drinking status**			
Current drinker	924 (3.655%)	Ref	
Ever drinker	3,871 (15.313%)	0.797 (0.650, 0.977)	0.029
Never drinker	20,484 (81.032%)	0.793 (0.659, 0.953)	0.013
**Family history of diabetes**			
No	24,654 (97.528%)	Ref	
Yes	625 (2.472%)	1.446 (1.191, 1.754)	< 0.001

Continuous variables were summarized as mean (SD) or medians (quartile interval); categorical variables were displayed as percentage (%).

FPG, fasting plasma glucose; BMI, body mass index; DBP, diastolic blood pressure; TC, total cholesterol, SBP, systolic blood pressure; TG triglyceride; ALT, alanine aminotransferase; TyG, the triglyceride-glucose index; TyG-BMI, triglyceride glucose-body mass index; BUN, blood urea nitrogen; LDL-c, low-density lipid cholesterol; Scr, serum creatinine, HDL-c, high-density lipoprotein cholesterol; AST aspartate aminotransferase.

**Figure 5:**
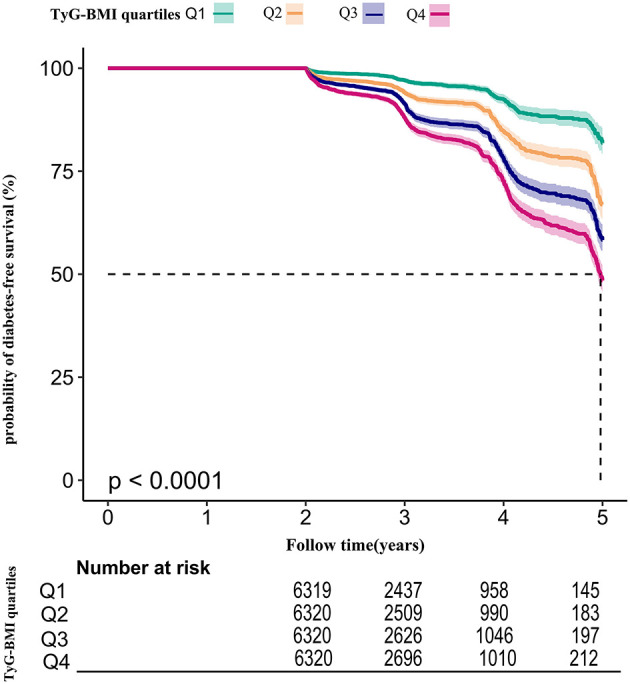
Kaplan–Meier event-free survival curve. Kaplan–Meier event-free survival curve. The probability of diabetes-free survival differed significantly between the TyG-BMI quartiles (log-rank test, *p* < 0.001). The probability of diabetes-free survival gradually decreased with increasing TyG-BMI, suggesting that the group with the highest TyG-BMI had the highest risk of diabetes.

Using the TyG-BMI quartile as stratification, Figure 5 presented Kaplan-Meier survival curves for diabetes-free survival probability. Among the quartiles of TyG-BMI, there were statistically significant differences in the probability of diabetes-free survival (log-rank test, *p* < 0.001). Prediabetic patients with the greatest TyG-BMI had the highest risk of progression to diabetes.

### The results of multivariable analyses using Cox proportional-hazards regression models

Three models were constructed using the Cox proportional-hazards regression model to investigate the association between the TyG-BMI and incident diabetes in patients with prediabetes (Table 4). An increase of 1 unit of TyG-BMI was associated with a 1.2% increase in diabetes risk in the crude model (HR = 1.012, 95%CI:1.011–1.013, *p* < 0.001). For participants with prediabetes, each additional unit of TyG-BMI increased their diabetes risk by 1.2% (HR = 1.012, 95% CI: 1.011–1.013) in the minimally adjusted model (Model I). As a result of the fully adjusted model (Model II), every 1 unit increase in TyG-BMI was associated with an increase in diabetes risk of 1.1% in participants with prediabetes (HR = 1.011, 95% CI 1.010–1.012). As evidenced by the confidence interval distribution, the model indicated a reliable relationship between TyG-BMI and diabetes risk in subjects with prediabetes.

**Table 4:** Relationship between TyG-BMI and the risk of diabetes in prediabetic patients in different models.

**Exposure**	**Crude model (HR, 95%CI)**	**Model I (HR, 95%CI) *P***	**Model II (HR, 95%CI) *P***	**Model III (HR, 95% CI) *P***
TyG-BMI	1.012 (1.011, 1.013) < 0.001	1.012 (1.011, 1.013) < 0.001	1.011 (1.010, 1.012) < 0.001	1.010 (1.009, 1.011) < 0.001
**TyG-BMI quartile**				
Q1	Ref	Ref	Ref	Ref
Q2	1.994 (1.710, 2.324) < 0.001	1.743 (1.494, 2.034) < 0.001	1.756 (1.502, 2.052) < 0.001	1.645 (1.405, 1.927) < 0.001
Q3	2.950 (2.553, 3.408) < 0.001	2.496 (2.156, 2.889) < 0.001	2.525 (2.174, 2.933) < 0.001	2.268 (1.943, 2.647) < 0.001
Q4	3.966 (3.449, 4.562) < 0.001	3.502 (3.038, 4.038) < 0.001	3.371 (2.906, 3.910) < 0.001	2.920 (2.497, 3.414) < 0.001
*P* for trend	1.514 (1.458, 1.572) < 0.001	1.477 (1.420, 1.536) < 0.001	1.451 (1.393, 1.511) < 0.001	1.387 (1.328, 1.449) < 0.001

Crude model: we did not adjust other covariates.

Model I: we adjusted age, sex.

Model II: we adjusted age, sex, SBP, DBP ALT, AST, BUN, LDL-C, HDL-c, family history of diabetes, drinking status, and smoking status.

Model III: we adjusted age (smooth), sex, SBP (smooth), DBP (smooth), ALT (smooth), AST (smooth), LDL-c (smooth), HDL-c (smooth), smoking status, drinking status, family history of diabetes.

HR, Hazard ratios; CI: confidence, Ref: reference.

### Sensitivity analysis

A series of sensitivity analyses were carried out to confirm the robustness of our conclusions. The TyG-BMI was first converted into quartile-based categorical variables, and the categorically modified TyG-BMI was then added back to the regression equation. The findings revealed that the effect sizes between groups were equidistant, and the trend of effect sizes was consistent with the result when the TyG-BMI was a continuous variable (Table 4).

Additionally, we introduced the continuity covariate as a curve into the equation using a GAM. As shown in Table 4, the outcome of Model III was reasonably consistent with the fully adjusted model (HR = 1.010, 95%CI: 1.009–1.011, *p* < 0.001).

Furthermore, we conducted a sensitivity analysis on participants who had never consumed alcohol (*n* = 20,484). After adjusting for confounding variables, the findings indicated that the TyG-BMI was also positively associated with the risk of diabetes (HR = 1.011, 95% CI:1.010–1.012, *p* < 0.001). We also excluded patients with a family history of diabetes for the sensitivity analyses. After adjusting for confounding variables, the results suggested that the TyG-BMI was still positively associated with diabetes risk in individuals with prediabetes (HR = 1.011, 95% CI:1.010–1.012, *p* < 0.001). In addition, restricting the analysis to participants with BMI < 25 kg/m^2^, the results suggested that the HR between the TyG-BMI and diabetes risk was 1.015 (95% CI:1.011–1.018, *P* < 0.001) (Table 5). Based on all the sensitivity analyses, it is evident that our findings were robust.

**Table 5:** Relationship between TyG-BMI and the risk of diabetes in participants with prediabetes in different sensitivity analyses.

**Exposure**	**Model I (HR, 95%CI) *P***	**Model II (HR, 95%CI) *P***	**Model III (HR, 95%CI) *P***
TyG-BMI	1.011 (1.010, 1.012) < 0.001	1.011 (1.010, 1.012) < 0.001	1.015 (1.011, 1.018) < 0.001
**TyG-BMI quartile**			
Q1	Ref	Ref	Ref
Q2	1.738 (1.466, 2.061) < 0.001	1.775 (1.513, 2.084) < 0.001	1.687 (1.431, 1.989) < 0.001
Q3	2.541 (2.158, 2.992) < 0.001	2.570 (2.204, 2.996) < 0.001	2.510 (2.076, 3.035) < 0.001
Q4	3.272 (2.776, 3.856) < 0.001	3.458 (2.970, 4.026) < 0.001	1.869 (1.187, 2.944) 0.007
*P*-for trend	< 0.001	< 0.001	< 0.001

Model I was a sensitivity analysis performed on participants who had never consumed alcohol (*N* = 20,484). We adjusted age, sex, SBP, DBP ALT, AST, BUN, LDL-C, HDL-c, family history of diabetes, and smoking status.

Model II was a sensitivity analysis performed on participants without a family history of diabetes (*N* = 24,654). We adjusted age, sex, SBP, DBP ALT, AST, BUN, LDL-C, HDL-c, drinking status, and smoking status.

Model III was a sensitivity analysis performed after excluding participants with BMI≥25 kg/m^2^ (*N* = 13,571). We adjusted age, sex, SBP, DBP ALT, AST, BUN, LDL-C, HDL-c, drinking status, smoking status, and family history of diabetes.

HR, Hazard ratios; CI, confidence; Ref, reference.

### Cox proportional hazards regression model with cubic spline functions to address non-linearity

**Figure 6:**
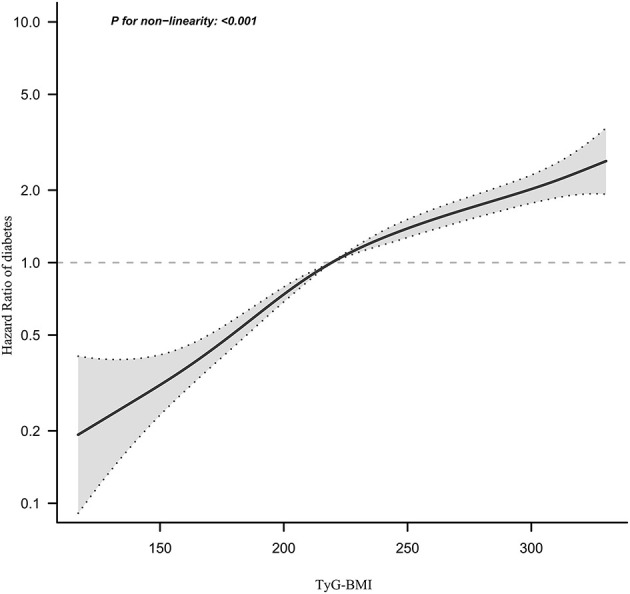
The non-linear relationship between TyG-BMI and the risk of diabetes in prediabetic patients. We used a Cox proportional hazards regression model with cubic spline functions to evaluate the relationship between the TyG-BMI and diabetes risk. The result showed that the relationship between the TyG-BMI and diabetes risk in prediabetic patients was non-linear, with the inflection point of the TyG-BMI ratio being 231.66.

We observed that the relationship between TyG-BMI and diabetes risk in prediabetic patients was non-linear using the Cox proportional hazards regression model with cubic spline functions (Figure 6). The *P*-value for the log-likelihood ratio test was < 0.001. We first determined that the inflection point of the TyG-BMI was 231.66 by the recursive algorithm and then used a two-piecewise Cox proportional hazards regression model to calculate the HR and CI for each side of the inflection point. Right before the inflection point, the HR was 1.007 (95% CI:1.005–1.009), while left after it was 1.017(95%CI:1.014–1.019) (Table 6).

**Table 6:** The result of two-piecewise linear regression model.

**Incident diabetes:**	**HR, 95%CI**	***P***
Fitting model by standard Cox regression	1.011 (1.010, 1.012)	< 0.001
**Fitting model by two-piecewise Cox regression**		
Inflection points of TyG-BMI	231.66
< 231.66	1.017 (1.014, 1.019)	< 0.001
≥231.66	1.007 (1.005, 1.009)	< 0.001
*P*-for log-likelihood ratio test		< 0.001

We adjusted age, sex, SBP, DBP ALT, AST, BUN, LDL-C, HDL-c, family history of diabetes, drinking status, and smoking status.

### The results of subgroup analyses

Interaction tests performed before **subgroup analyses** showed that age and SBP interacted with TyG-BMI (*P* < 0.001). In contrast, sex, smoking, and alcohol consumption did not interact with TyG-BMI (*P* > 0.05) (Supplementary Table S2). Therefore, further subgroup analyses with prespecified or exploratory age and SBP were performed (Table 7). However, there was no significant interaction of SBP as a categorical variable with TyG-BMI (*P* for interaction >0.05). The results showed that in the age subgroup, the interaction between TyG-BMI and age was significant (*P* for interaction < 0.001). Specifically, a stronger relationship between TyG-BMI and diabetes risk was observed in participants aged < 50 years. Among participants aged < 30 years, 30–40 years, and 40–50 years, the HRs for the association between TyG-BMI and the risk of diabetes in prediabetic patients were 1.020, 1.019, and 1.016, respectively (all *P* < 0.001). In contrast, a weaker relationship between TyG-BMI and diabetes risk was observed in prediabetic participants aged >50 years. Among participants aged 50–60, 60–70, and ≥70, the HRs for the relationship between TyG-BMI and the risk of diabetes was 1.009, 1.006, and 1.008, respectively (all *P* < 0.001).

**Table 7:** Stratified associations between TyG-BMI and diabetes in participants with prediabetes by age and SBP.

**Characteristic**	**No of participants**	**HR (95%CI)**	***P*-value**	***P*-for interaction**
Age (years)				< 0.001
< 30	1,464	1.020 (1.011, 1.029)	< 0.001	
30 to < 40	6,055	1.019 (1.017, 1.022)	< 0.001	
40 to < 50	5,514	1.016 (1.014, 1.019)	< 0.001	
50 to < 60	5,907	1.009 (1.007, 1.011)	< 0.001	
60 to < 70	4,278	1.006 (1.003, 1.008)	< 0.001	
≥70	2,061	1.008 (1.005, 1.011)	< 0.001	
SBP (mmHg)				0.2484
< 140	19,902	1.012 (1.010, 1.013)	< 0.001	
≥140	5,377	1.010 (1.008, 1.012)	< 0.001	

Above model adjusted for age, sex, SBP, DBP ALT, AST, BUN, LDL-c, HDL-c, family history of diabetes, drinking status, and smoking status.

In each case, the model is not adjusted for the stratification variable.

HR, Hazard ratios; CI, confidence; Ref, reference.

### The results of the ROC curve analysis

**Figure 7:**
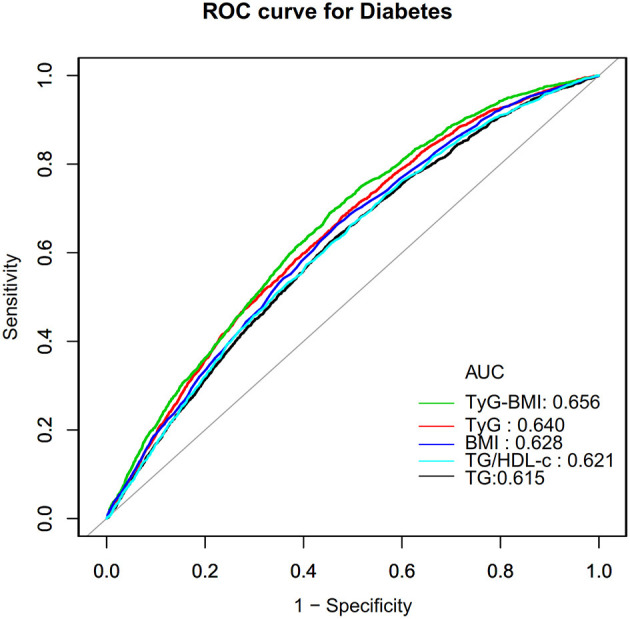
The results of ROC curve analysis for measuring the ability of TyG-BMI, BMI, TG, TyG, and TG/HDL-c ratio to predict the risk of diabetes.

In addition, we drew a ROC curve to measure the ability of TyG-BMI, BMI, TyG, TG, and TG/HDL-c ratio to predict the risk of diabetes (Figure 7). The areas under the curve of each variable were as follows: TG: 0.615 < TG/HDL-c ratio:0.621 < BMI: 0.628 < TyG: 0.640 < TyG-BMI:0.656. The highest Youden index of TG, TG/HDL-c ratio, BMI, TyG, and TyG-BMI was 0.1670, 0.1700,0.1954, 0.2030, 0.2513, and the corresponding optimal cut-off value was 1.595, 1.1463, 24.760, 8.808, 220.238, respectively. The Youden index and AUC of TyG-BMI were the biggest, so the predictive ability of TyG-BMI to incident diabetes was better than that of other variables (Supplementary Table S3).

## Discussion

In this retrospective cohort study, we examined the link between the TyG-BMI and diabetes risk in patients with prediabetes. We found that a higher TyG-BMI was linked to a significantly higher risk of diabetes among patients with prediabetes. Additionally, an inflection point was identified, and different relationships between the TyG-BMI and diabetes risk were detected on both sides.

A Japanese study found an 8.5% progression rate from prediabetes to diabetes within five years (29). Americans with prediabetes aged 70–79 have a 10.6% chance of progressing to diabetes over the next seven years (30). Participants with prediabetes in the present study had a cumulative incidence of diabetes of 10.63% over a median follow-up of 2.89 years. Participants' age, follow-up time, and ethnicity may be responsible for these differences in diabetes incidence among these patients. It is worth noting that all studies have shown that patients with prediabetes are at high risk for developing diabetes. Therefore, it is crucial to actively identify various other risk factors for progressing to diabetes from prediabetes.

The TyG index is a combined marker containing FPG and TG and is considered an alternative marker of IR (14, 31). In recent years, TyG-BMI, a metric that combines BMI with TyG, has emerged as a new obesity-related parameter. Studies have shown that TyG-BMI is a better predictor of IR than traditional lipids, BMI, and TyG index (16). Furthermore, several recent studies have found that TyG-BMI is strongly associated with non-alcoholic fatty liver, stroke, prehypertension, and diabetes (32–34). However, the connection between the TyG-BMI index and the prevalence of diabetes has received little attention with only two research covering the subject. 116,661 participants in a cohort study had their physicals examined. Multivariate Cox regression analysis revealed an independent relationship between the TyG-BMI index and new-onset diabetes in the general population (HR 1.50/standard deviation increase, 95% CI: 1.40 to 1.60, *P* < 0.0001) (18). Another cross-sectional study conducted in Spain supported this conclusion. According to results from multivariate-adjusted models, participants in the fourth quartile of the TyG-BMI index had a 3.63-fold higher risk of getting diabetes than those in the first quartile (17). Our study complemented the existing literature, which supported the hypothesis that an elevated TyG-BMI index is positively associated with the risk of new-onset diabetes. In contrast to earlier studies, the independent variables in our study used both the TyG-BMI index as a categorical variable and a continuous variable to explore their relationship with diabetes risk, thus reducing information loss and quantifying the relationship between them. Second, to the best of our knowledge, this is the first study to examine the association between the TyG-BMI and diabetes risk in individuals with prediabetes, a population with a high propensity to develop diabetes. Identifying TyG-BMI as a risk factor for progression from prediabetes to diabetes and clarifying the association between them would be beneficial for diabetes prevention in patients with prediabetes.

In addition, based on the population with prediabetes, our previous study identified an important lipid index, TG/HDL-c ratio, as an important risk predictor for diabetes (35). Therefore, we constructed ROC curves to estimate the ability of TyG-BMI, BMI, TyG, TG, and TG/HDL-c ratio to predict the risk of progression to diabetes in patients with prediabetes. We found that the AUC and highest Youden index of the TyG-BMI index fared better than any other component of the TyG-BMI and TG/HDL-c ratio. This finding shows that TyG-BMI may be a valuable marker for predicting the onset of diabetes in patients with prediabetes. These indicators provide significant risk predictors for future risk prediction models for the progression of prediabetes to diabetes. Furthermore, sensitivity analyses found that their association persisted in prediabetic patients with a BMI < 25 kg/m^2^, no family history of diabetes, and no alcohol consumption. This study encourages clinical consultation and provides a reference for enhancing diabetes prevention in individuals with prediabetes.

The underlying mechanisms relating TyG-BMI to diabetes risk remain unclear, but it may be associated with IR. Research has confirmed that IR plays a crucial role in diabetes occurrence and progression (36). TyG-BMI represents a combination of FPG, TG, and BMI. FPG levels are a reflection of insulin sensitivity in the liver and insulin secretion by the pancreas (37). A higher level of FPG is associated with an increased risk of diabetes among people with normal FPG levels (38). In addition, the role of BMI and TG in identifying IR has been well established in previous studies (39–41). Therefore, the underlying mechanism of the relationship between TyG-BMI and the risk of developing diabetes may be related to the association of three factors, FPG, TG, and BMI, with IR.

Furthermore, our study observed a non-linear relationship between the TyG-BMI and diabetes risk in individuals with prediabetes for the first time. After controlling for confounders, the TyG-BMI inflection point was 231.66. When the TyG-BMI was < 231.66, each unit increase was associated with a 1.7% increase in the risk of diabetes. A 1-unit increase in TyG-BMI was associated with a 0.7% increase in the risk of diabetes when the TyG-BMI was >231.66. It could be found that compared to participants with a TyG-BMI>231.66, those with TyG-BMI ≤ 231.66 generally are younger and have lower DBP, LDL-c, AST, SBP, and ALT. In addition, those with a TyG-BMI ≤ 231.66 had a lower proportion of currently drinking and smoking (Supplementary Table S4). However, these indicators are strongly linked to incident diabetes (42–46). Because of the presence of these risk factors, when the TyG-BMI was >231.66, the TyG-BMI had a relatively weak effect on diabetes. On the contrary, among those with a TyG-BMI of < 231.66, these diabetes risk factors were lower, the impact on diabetes was lower, and the effect of TyG-BMI was relatively enhanced. Furthermore, non-linear relationships are those in which the change in one variable does not correspond to the same constant change in the other variable. In other words, it could imply that the relationship between two variables is either non-existent or unpredictable. Non-linear entities, on the other hand, can be related to each other in predictable but more complex ways than linear ones. Because of the complexities of the relationship between TyG-BMI and diabetes risk, the non-linear relationship may be closer to the true relationship. The discovery of a curvilinear relationship between the TyG-BMI and incident diabetes in prediabetic patients has significant clinical implications. It serves as a resource for promoting clinical consultation and optimizing diabetes prevention decision-making in patients with prediabetes.

Subgroup analysis revealed some interesting findings in this study. Young adults have a higher risk of diabetes associated with their TyG-BMI than other age groups. After further analysis of the baseline information of the study population grouped according to age, young-aged people (< 50 years old) were found had lower ALT, DBP, LDL-c, SBP, and a lower proportion of currently drinking (Supplementary Table S5). Therefore, the level of these risk factors for diabetes was lower in young adults, the impact on diabetes was reduced, and the effect of the TyG-BMI was relatively enhanced.

Several strengths are worthy of attention in this investigation. (i)This is the first time Chinese individuals with prediabetes have been employed as a research population to explore the association between TyG-BMI and diabetes risk. (ii) We elucidated the non-linear association between TyG-BMI and diabetes risk and identified the inflection point. This is a great improvement compared to other previous studies. (iii) Multiple imputations were used to account for missing data. This strategy maximizes statistical power while minimizing the bias caused by missing covariate data. (iv) To ensure the robustness of the conclusions, a series of sensitivity analyses were conducted, including converting TyG-BMI into a categorical variable, using a GAM to insert the continuity covariate into the equation as a curve, and reanalyzing the association between TyG-BMI and incident diabetes after excluding alcohol and cigarette consumers as well as participants with a family history of diabetes.

Nonetheless, the following restrictions should be noted: (i) Because all of the participants were Chinese, additional studies are needed to assess the association between this new risk marker TyG-BMI and the risk of progression from prediabetes to diabetes. In the future, we will collaborate with investigators outside of China to validate their association in other populations of different genetic backgrounds. (ii) Diabetes was defined as a fasting plasma glucose (FPG) level of 7.00 mmol/L and/or self-reported diabetes during the follow-up period, but not a measurement of glycosylated hemoglobin or a 2-h oral glucose tolerance test. Consequently, the incidence of diabetes may be understated. (iii) Certain indications related to diabetes and IR were absent from the raw data, including waist-to-hip ratio, waist circumference, and insulin concentration. In addition, the present investigation only evaluated TG, FPG, BMI, and other parameters at baseline; TyG-BMI variations over time were not included. In the future, we may seek to construct our studies or partner with other researchers to collect as many factors as feasible, including TyG-BMI change information over time. (iv) The type of diabetes cannot be determined. Type 2 diabetes mellitus (T2DM) is the most prevalent form of diabetes in China, accounting for more than 90% of all diabetes cases (47). Consequently, our results are representative of T2DM. Finally, this retrospective observational study did not demonstrate a causal relationship between the TyG-BMI and the risk of diabetes in patients with prediabetes; rather, it established an association.

## Conclusion

This study demonstrates a positive and non-linear relationship between the TyG-BMI and the risk of incident diabetes in Chinese adults with prediabetes. When the TyG-BMI was < 231.66, there was a significant positive association with the risk of progression from prediabetes to diabetes. The present study offers more references to promote clinical consultation and to optimize diabetes prevention decisions for patients with prediabetes.

## Data availability statement

The original contributions presented in the study are included in the article/Supplementary material, further inquiries can be directed to the corresponding author/s.

## Ethics statement

The studies involving human participants were reviewed and approved by the Rich Healthcare Group Review Board. Written informed consent for participation was not required for this study in accordance with the national legislation and the institutional requirements. Written informed consent was not obtained from the individual(s) for the publication of any potentially identifiable images or data included in this article. No identifiable images data statement are presented in this manuscript.

## Author contributions

YH, HH, and QL conceived of the study, conducted statistical analysis, and drafted the manuscript. ZD and DL revised the manuscript and designed the study. All authors have reviewed and approved the final version of the text.
