# Supplementary material for: Triglyceride glucose-body mass index and the risk of progression to diabetes from prediabetes: A 5-year cohort study in Chinese adults
Source: Front Public Health. 2023 Feb 3;11:1028461. doi: 10.3389/fpubh.2023.1028461 (PMC9935616; doi:10.3389/fpubh.2023.1028461)
Supplement: Supplementary file 1 [file Table_1.DOCX]

**Triglyceride glucose-body mass index and the risk of progression to diabetes from prediabetes: a 5-year cohort study in Chinese adults**

**Running title: TyG-BMI and diabetes**

Yong Han^1#^, Haofei Hu^2#^, Qiming Li ^1^^#^, Zhe Deng^1*^, Dehong Liu^1*^

^1^ Department of Emergency, Shenzhen Second People's Hospital, Shenzhen 518035, Guangdong Province, China

^2^ Department of Nephrology, Shenzhen Second People's Hospital, Shenzhen 518035, Guangdong Province, China

Yong Han^1#^, Haofei Hu^2#^, and Qiming Li ^1#^ have contributed equally to this work.

*Corresponding author

Zhe Deng

Department of Emergency, Shenzhen Second People's Hospital

No.3002 Sungang Road, Futian District,

Shenzhen 518035,

Guangdong Province,

China.

[anyo511023@163.com](mailto:anyo511023@163.com)

*Corresponding author

Dehong Liu

Department of Emergency, Shenzhen Second People's Hospital

No.3002 Sungang Road, Futian District,

Shenzhen 518035,

Guangdong Province,

China

E-mail: dhliu_emergency@163.com

**Table S1 collinearity screening**

|  | Step 1 | Step 2 |
| --- | --- | --- |
| TyG-BMI | 1.8 | 1.8 |
| Age(years) | 1.3 | 1.3 |
| Sex | 2.2 | 2.2 |
| SBP (mmHg) | 1.9 | 1.9 |
| DBP (mmHg) | 1.8 | 1.8 |
| HDL-c(mmol/L) | 1.3 | 1.1 |
| LDL-c(mmol/L) | 4.3 | 1.1 |
| ALT(U/L) | 3.5 | 3.5 |
| AST(U/L) | 3.1 | 3.1 |
| BUN (mmol/L) | 1.2 | 1.2 |
| TC(mmol/L) | 5 | NA |
| Scr (μmol/L) | 1.8 | 1.8 |
| Smoking status | 1.3 | 1.3 |
| Drinking status | 1.2 | 1.2 |
| Family history of diabetes | 1 | 1 |

NA was the excluded variable

Abbreviations: BMI, body mass index; DBP, diastolic blood pressure; SBP, systolic blood pressure; TG triglyceride, TC, total cholesterol, TyG-BMI, triglyceride glucose-body mass index; LDL-C,low-density lipid cholesterol; HDL-c, high-density lipoprotein cholesterol; AST aspartate aminotransferase; ALT,alanine aminotransferase; BUN, blood urea nitrogen; Scr, serum creatinine.

**Table S2 Variables included age, sex, SBP, DBP, smoking status and drinking status interacted with TyG-BMI**

| Characteristic |  | *P*-value |
| --- | --- | --- |
| Age |  | **<0.001** |
| Sex |  | **0.1125** |
| SBP (mmHg) |  | **<0.001** |
| Drinking status |  | 0.586 |
| Smoking status |  | 0.056 |

Note 1: *P*>0.05 suggested that there was no interaction

Note 2: Above model adjusted for age, sex, SBP, DBP ALT, AST, BUN, LDL-c, HDL-c, family history of diabetes, drinking status, and smoking status.

Note 3: In each case, the model is not adjusted for the stratification variable

**Table S3. AUC with the 95% CI of TyG-BMI, BMI, TyG, and TG for predicting DM**

| Test | AUC(95%CI) | Best threshold | Specificity | Sensitivity | Yorden Index |
| --- | --- | --- | --- | --- | --- |
| BMI | 0.6281(0.6172-0.6390) | 24.7600 | 0.5313 | 0.6641 | 0.1954 |
| TG | 0.6153(0.6042-0.6042) | 1.5950 | 0.5936 | 0.5734 | 0.1670 |
| TyG | 0.6405(0.6297-0.6512) | 8.8076 | 0.5203 | 0.6828 | 0.2030 |
| TyG-BMI | 0.6560(0.6455-0.6666) | 220.2382 | 0.5466 | 0.6902 | 0.2513 |
| TG/HDL-c ratio | 0.6211(0.6012-0.6324) | 1.1463 | 0.5462 | 0.6283 | 0.1700 |

AUC: area under the curve; BMI, body mass index; TG, Triglyceride; TyG, the triglyceride-glucose index; TyG-BMI, triglyceride glucose-body mass index; TG/HDL-C ratio, triglyceride-to-high density lipoprotein cholesterol ratio CI: Confidence interval.

**Table S4 The Baseline Characteristics of participants on both sides of the inflection point(231.66)**

| TyG-BMI | <231.66 | >=231.66 | Standardize diff. | P-value |
| --- | --- | --- | --- | --- |
| N | 16134 | 9145 |  |  |
| Age(years) | 48.56 ± 14.21 | 50.58 ± 13.00 | 0.15 (0.12, 0.17) | <0.001 |
| BMI (kg/m^2^) | 22.98 ± 2.25 | 27.89 ± 2.26 | 2.18 (2.15, 2.21) | <0.001 |
| SBP (mmHg) | 124.60 ± 17.18 | 131.69 ± 17.38 | 0.41 (0.38, 0.44) | <0.001 |
| DBP (mmHg) | 76.39 ± 10.63 | 81.89 ± 11.14 | 0.50 (0.48, 0.53) | <0.001 |
| HDL-c(mmol/L) | 1.36 ± 0.31 | 1.27 ± 0.29 | 0.30 (0.27, 0.32) | <0.001 |
| LDL-c(mmol/L) | 2.83 ± 0.71 | 2.99 ± 0.74 | 0.22 (0.19, 0.24) | <0.001 |
| ALT(U/L) | 23.03 ± 18.01 | 37.57 ± 27.99 | 0.62 (0.59, 0.64) | <0.001 |
| AST(U/L) | 24.20 ± 10.23 | 30.08 ± 13.66 | 0.49 (0.46, 0.51) | <0.001 |
| BUN (mmol/L) | 4.95 ± 1.26 | 5.06 ± 1.24 | 0.08 (0.06, 0.11) | <0.001 |
| Sex |  |  | 0.39 (0.37, 0.42) | <0.001 |
| Male | 9633 (59.71%) | 7101 (77.65%) |  |  |
| Female | 6501 (40.29%) | 2044 (22.35%) |  |  |
| Smoking status |  |  | 0.26 (0.23, 0.28) | <0.001 |
| Current drinker | 3033 (18.80%) | 2682 (29.33%) |  |  |
| Ever | 613 (3.80%) | 421 (4.60%) |  |  |
| Never | 12488 (77.40%) | 6042 (66.07%) |  |  |
| Drinking status |  |  | 0.16 (0.14, 0.19) | <0.001 |
| Current drinker | 456 (2.83%) | 468 (5.12%) |  |  |
| Ever drinker | 2261 (14.01%) | 1610 (17.61%) |  |  |
| Never drinker | 13417 (83.16%) | 7067 (77.28%) |  |  |
| Family history of diabetes |  |  | 0.00 (-0.02, 0.03) | 0.873 |
| No | 15737 (97.54%) | 8917 (97.51%) |  |  |
| Yes | 397 (2.46%) | 228 (2.49%) |  |  |

Abbreviations: TyG-BMI, triglyceride glucose-body mass index; DBP, diastolic blood pressure; SBP, systolic blood pressure; LDL-C,low-density lipid cholesterol; HDL-c, high-density lipoprotein cholesterol; AST aspartate aminotransferase; ALT,alanine aminotransferase; BUN, blood urea nitrogen;

**Table S5 Comparison of baseline characteristics of participants younger than 50 and older than 50**

| Age(years) | <50 | >=50 | Standardize diff. | P-value |
| --- | --- | --- | --- | --- |
| N | 13033 | 12246 |  |  |
| Age(years) | 38.03 ± 6.54 | 61.28 ± 8.37 | 3.10 (3.06, 3.13) | <0.001 |
| BMI (kg/m^2^) | 24.52 ± 3.47 | 25.02 ± 3.01 | 0.15 (0.13, 0.18) | <0.001 |
| SBP (mmHg) | 122.53 ± 15.23 | 132.10 ± 18.56 | 0.56 (0.54, 0.59) | <0.001 |
| DBP (mmHg) | 76.76 ± 10.76 | 80.09 ± 11.27 | 0.30 (0.28, 0.33) | <0.001 |
| HDL-c(mmol/L) | 1.32 ± 0.29 | 1.34 ± 0.32 | 0.09 (0.06, 0.11) | <0.001 |
| LDL-c(mmol/L) | 2.77 ± 0.70 | 3.01 ± 0.73 | 0.33 (0.30, 0.35) | <0.001 |
| ALT(U/L) | 30.72 ± 26.57 | 25.70 ± 18.67 | 0.22 (0.19, 0.24) | <0.001 |
| AST(U/L) | 25.97 ± 12.70 | 26.71 ± 11.03 | 0.06 (0.04, 0.09) | <0.001 |
| BUN (mmol/L) | 4.77 ± 1.17 | 5.22 ± 1.29 | 0.37 (0.34, 0.39) | <0.001 |
| Sex |  |  | 0.07 (0.05, 0.10) | <0.001 |
| Male | 8844 (67.86%) | 7890 (64.43%) |  |  |
| Female | 4189 (32.14%) | 4356 (35.57%) |  |  |
| Smoking status |  |  | 0.14 (0.11, 0.16) | <0.001 |
| Current smoker | 2600 (19.95%) | 3115 (25.44%) |  |  |
| Ever smoker | 594 (4.56%) | 440 (3.59%) |  |  |
| Never smoker | 9839 (75.49%) | 8691 (70.97%) |  |  |
| Drinking status |  |  | 0.16 (0.13, 0.18) | <0.001 |
| Current drinker | 349 (2.68%) | 575 (4.70%) |  |  |
| Ever drinker | 2280 (17.49%) | 1591 (12.99%) |  |  |
| Never drinker | 10404 (79.83%) | 10080 (82.31%) |  |  |
| Family history of diabetes |  |  | 0.08 (0.06, 0.10) | <0.001 |
| No | 12633 (96.93%) | 12021 (98.16%) |  |  |
| Yes | 400 (3.07%) | 225 (1.84%) |  |  |

Abbreviations: ; BMI, body mass index;; DBP, diastolic blood pressure; SBP, systolic blood pressure; LDL-C,low-density lipid cholesterol; HDL-c, high-density lipoprotein cholesterol; AST aspartate aminotransferase; ALT,alanine aminotransferase; BUN, blood urea nitrogen;

Table S6 Association between TyG-BMI and diabetes risk in prediabetic patients in multivariable-adjusted Cox proportional hazards regression based on original data without multiple interpolation.

| Exposure | Crude model (HR,95%CI) | Model I(HR,95%CI) P |
| --- | --- | --- |
| TyG-BMI | 1.012 (1.011, 1.013) <0.001 | 1.013 (1.008, 1.018) <0.001 |
| TyG-BMI quartile | |  |
| Q1 | Ref | Ref |
| Q2 | 2.051 (1.764, 2.385) | 2.136 (1.038, 4.397) 0.039 |
| Q3 | 3.010 (2.611, 3.471) <0.001 | 4.065 (2.037, 8.111) <0.001 |
| Q4 | 4.060 (3.537, 4.659) <0.001 | 4.559 (2.283, 9.106) <0.001 |

Crude model: we did not adjust other covariates

Model I: we adjusted age, sex, SBP, DBP ALT, AST, BUN, LDL-C, HDL-c, family history of diabetes, drinking status, and smoking status.

**FigureS1. Data visualization of TyG-BMI of all participants from the diabetes and non-diabetes groups.**
