# Supplementary figures and images for: Triglyceride glucose-body mass index and the risk of progression to diabetes from prediabetes: A 5-year cohort study in Chinese adults
Source: Front Public Health. 2023 Feb 3;11:1028461. doi: 10.3389/fpubh.2023.1028461 (PMC9935616; doi:10.3389/fpubh.2023.1028461)

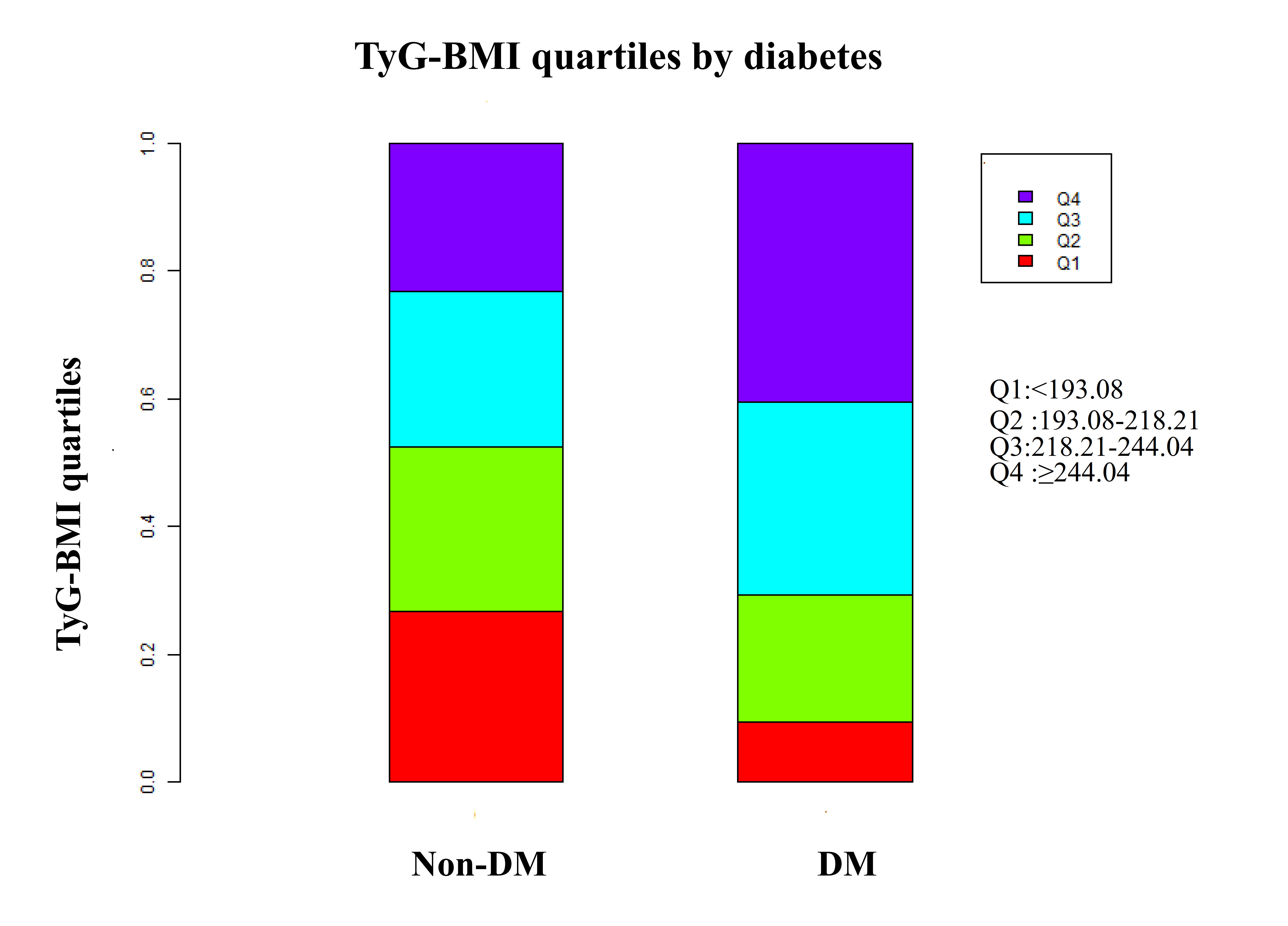

Supplement: Supplementary file 3 [file Image_1.TIF]
